# Salamander Insights Into Ageing and Rejuvenation

**DOI:** 10.3389/fcell.2021.689062

**Published:** 2021-06-07

**Authors:** Maximina H. Yun

**Affiliations:** ^1^CRTD/Center for Regenerative Therapies Dresden, Technische Universität Dresden, Dresden, Germany; ^2^Max Planck Institute of Molecular Cell Biology and Genetics, Dresden, Germany

**Keywords:** axolotl, newt, cellular senescence, negligible senescence, regeneration, cancer

## Abstract

Exhibiting extreme regenerative abilities which extend to complex organs and entire limbs, salamanders have long served as research models for understanding the basis of vertebrate regeneration. Yet these organisms display additional noteworthy traits, namely extraordinary longevity, indefinite regenerative potential and apparent lack of traditional signs of age-related decay or “negligible senescence.” Here, I examine existing studies addressing these features, highlight outstanding questions, and argue that salamanders constitute valuable models for addressing the nature of organismal senescence and the interplay between regeneration and ageing.

## Introduction

Salamanders, or urodele amphibians, stand out among vertebrates due to their ability to regenerate extensive sections of their body plan including ocular tissues, jaws, lungs, sections of their heart and brain, spinal cord, and entire limbs throughout their lives (Brockes and Kumar, 2008; Cox et al., 2019). As the evolutionarily closest organisms to humans capable of complex regeneration, salamanders constitute valuable models for regenerative biology studies. In particular, the axolotl—*Ambystoma mexicanum—*and the Iberian ribbed newt—*Pleurodeles waltl—*are two laboratory-tractable systems whose adoption has exponentially grown in recent years due to the ease of captive breeding and rearing (Khattak et al., 2014; Joven et al., 2015), efficient transgenesis and genome editing methods (Khattak et al., 2013b; Hayashi et al., 2014; Fei et al., 2018; Cai et al., 2019), availability of genomic and transcriptomic information (Elewa et al., 2017; Nowoshilow et al., 2018; Smith et al., 2019), and advanced imaging techniques (Masselink and Tanaka, 2020; Subiran Adrados et al., 2020; Box 1). Newts present a conventional salamander life cycle, undergoing metamorphosis and becoming fully developed adults with reduced to imperceptible continuous growth. In contrast, axolotls are neotenic organisms, exhibiting larval traits throughout their lives and indefinite growth. While occasional differences in regenerative capacity (Suetsugu-Maki et al., 2012) and mechanisms (Tanaka, 2016; Tanaka et al., 2016) exist, both species are capable of extensive organ and appendage regeneration following important clade-conserved principles. Particularly, salamander regeneration is associated with an unusual ability to regulate the plasticity of the differentiated state. Instead of relying exclusively on stem cells, the progenitors for the new structure are often obtained through limited reprogramming—dedifferentiation and transdifferentiation*—*of mature, differentiated adult cells (Tanaka and Reddien, 2011; Yun et al., 2013, 2014). In the context of the axolotl limb, the connective tissue cells at the stump dedifferentiate to form the various connective tissue derivatives of the new structure (Gerber et al., 2018). In newts, muscle regeneration relies on progenitors derived from dedifferentiation of mature muscle fibres (Lo et al., 1993; Tanaka et al., 2016), while the lens of the eye is regenerated *de novo* through transdifferentiation of pigmented epithelial cells of the dorsal iris (Henry and Tsonis, 2010). Reversals of the differentiated state for the generation of regenerative progenitors are also common in other vertebrates capable of complex regeneration, such as zebrafish (Jopling et al., 2010; Knopf et al., 2011), yet rarely observed in mammals. In this connection, the existence of roadblocks to dedifferentiation has been proposed to underlie the limited regenerative potential found in mammalian systems (Pajcini et al., 2010; Yun et al., 2013).

**Experimental toolbox for salamander models^∗^.**

• **Germline transgenesis.** Tools for germline transgenesis are available for both axolotls and Iberian ribbed newts, based on I-*Sce*I meganuclease and Tol2 transposon technologies, including the CRE/LoxP system for tissue and time dependent control of gene expression (Khattak et al., 2013a; Hayashi and Takeuchi, 2015).
• **Genome assembly and CRISPR-mediated gene editing.** The recent sequencing and assembly of the 32-Gb axolotl genome (Nowoshilow et al., 2018; Smith et al., 2019) and the 20-Gb *P. waltl* genome (Elewa et al., 2017) provides a rich platform for investigations into the molecular basis of biological phenomena. Together with TALEN and CRISPR/Cas9-mediated gene editing (Khattak et al., 2013a; Hayashi et al., 2014; Fei et al., 2018; Cai et al., 2019), it is possible to assess candidate genes for functional analysis.
• **Somatic gene delivery methods.** Various technologies are available for gene delivery to salamander cells and tissues (Echeverri and Tanaka, 2003; Yun et al., 2013), including electroporation and viral transfection methods (Khattak et al., 2013a; Whited et al., 2013; Oliveira et al., 2018).
• **Cell and tissue transplantation.** The amenability of salamanders to cell and tissue transplantation combined with transgenic technologies (Kragl et al., 2009; Kragl and Tanaka, 2009; Lopez et al., 2014; Yun et al., 2015), has been informative toward understanding key aspects of development and regeneration.
• **Advanced imaging.** Many salamander tissues are optically transparent, and highly suited to live imaging. Further, several optical clearing methods have been adapted to the salamander system, enabling volumetric quantitative imaging (Duerr et al., 2020; Masselink and Tanaka, 2020; Pinheiro et al., 2020; Subiran Adrados et al., 2020).
• **Chemical screenings.** Due to their size and skin mediated compound exchange, salamanders can be used for moderate-throughput screening of pharmaceutical compounds (Ponomareva et al., 2015).

^∗^Adapted from Yu and Yun (2020).

## Indefinite Regenerative Capacity

A salient feature of salamander regeneration is its resilience. Urodele regenerative capacity does not decline with time, and most studies suggest it is not impaired by repetitive regeneration events (Yun, 2015). A landmark study by Eguchi et al. (2011) tracked the process of lens regeneration over 16 years in Japanese newts, removing the lens from the same animals 18 times and allowing them to undergo regeneration. Remarkably, the resulting lenses were structurally identical to the original ones and expressed similar levels of lens-specific genes. Subsequent analysis revealed that the transcriptomes of young and old (19-times regenerated) lenses are nearly indistinguishable (Sousounis et al., 2015), showcasing the robustness of newt lens regeneration. Of note, by the end of the study the specimens were at least 30 years old, representing a geriatric population in this species (Eguchi et al., 2011). This provides an interesting contrast to the declines in regenerative capacities observed in most vertebrate contexts (Yun, 2015). Additional studies indicate that repetitive amputations do not affect tail regenerative potential in the newt *Triturus carnifex*, as examined over a 10 year period with up to nine tail regeneration cycles (Margotta et al., 2002; Margotta, 2008), nor that of the axolotl limb, challenged by five regeneration rounds during 3 years (Yun et al., 2015). In this connection, a recent study observed increasing rates of incomplete or failed regeneration after 3 regenerative cycles in the axolotl (Bryant et al., 2017). This interesting observation was based on studies using American axolotl strains, whereas similar studies in European strains have not shown the described regenerative impairment. It is thus conceivable that the phenotypic differences stem from a diverse genetic background, something which should be addressed by further studies. Taken together, the evidence to date suggests that the ability of urodeles to regenerate complex structures does not decline with time or serial regeneration cycles. In mammals, loss of regenerative potential with ageing has been largely attributed to the ageing of stem cell populations and/or their niche (Yun, 2015). Whether the prevalence of dedifferentiation as a regenerative mechanism in salamanders is linked to the indefinite nature of their regenerative potential remains an outstanding question.

## Extreme Lifespans

**FIGURE 1:**
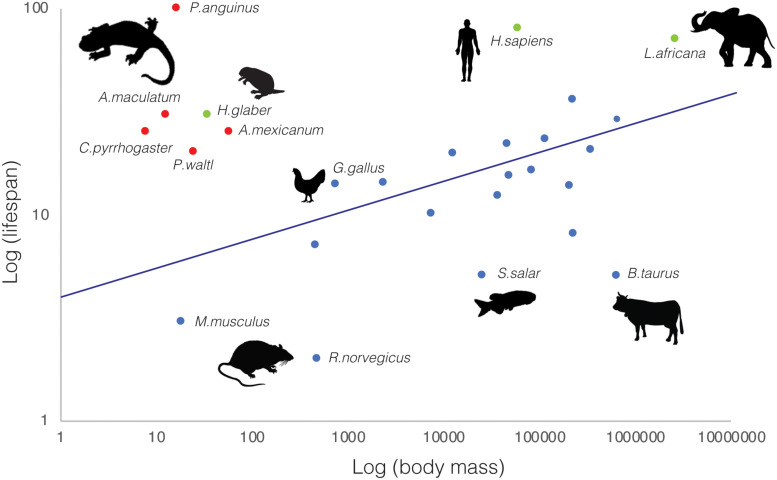
Salamanders are lifespan outliers. Relationship between average body mass (g) and lifespan (years) for selected salamander (red) and representative vertebrate species (blue). Additional upper-end lifespan outliers (naked mole rat—*Heterocephalus glaber*-, African bush elephant—*Loxodonta africana—*and human—*Homo sapiens*—) are indicated in green. Animal silhouettes (not drawn to scale) represent the vertebrate clades to which the selected representative species belong to. Data was obtained from various sources, including ADW Animal diversity web, AnAge (Tacutu et al., 2018) and Amniote Life History Database (Myhrvold et al., 2015).

Beyond their remarkable regenerative abilities, salamanders exhibit extraordinary longevity (Sousounis et al., 2014), constituting lifespan outliers with respect to organismal size (Figure 1). Among animal species, there is a notable correlation between body mass and lifespan, with larger animals living longer. Yet, salamanders break this rule by several orders of magnitude. For example, axolotls—average mass: 60–110 g*—*live over 20 years (CRTD colony and (Warburg, 2007)), *P. walt* newts—average mass: 25 g*—*live up to 20 years in the wild (Warburg, 2007; Tacutu et al., 2018), Japanese newts—*Cynops pyrrhogaster*, average mass: 8 g*—*have a 25 year lifespan (Sousounis et al., 2015), spotted salamanders—*Ambystoma maculatum*; average mass: 13 g*—*reach 30 years of age, and cave olms—*Proteus anguinus*; average mass: 17 g—can surpass 100 years (Voituron et al., 2011; Tacutu et al., 2018). Indeed, they match and in some cases exceed the lifespan/body mass ratios found in other well-known outliers such as the naked mole rat (Ruby et al., 2018) and Brandt’s bat (Seim et al., 2013). This is even more remarkable given that most salamander longevity data derive from specimens in the wild (Warburg, 2007), where animals are exposed to environmental challenges, predation, pathogens, and food source fluctuations. The establishment of research colonies for certain species, enabling breeding and rearing of individuals under controlled conditions, has contributed to the acquisition of more accurate lifespan measurements. Unsurprisingly, in most cases these surpass the estimates obtained from wild specimens, as in the extreme case of *P. anguinus*, whose lifespan fluctuates from 15 years in the wild to a predicted maximum exceeding 100 years in lab cave conditions established in the 1950’s (Voituron et al., 2011; Tacutu et al., 2018). Thus, salamanders are not only lifespan outliers, but also in many cases their longevity may be underestimated.

## Negligible Senescence

While the underlying basis of their exceptional longevity remains unknown, salamanders exhibit an uncommon resistance to ageing. Although few studies have addressed this topic, these, together with evidence from captive records in zoos and laboratories, suggest that a number of urodele species do not display the traditional signs of physiological decay that accompany mammalian ageing and are thus considered organisms of “negligible senescence” (Finch, 1990; Margotta et al., 2002; Cayuela et al., 2019). This phenomenon, also observed in other vertebrates such as turtles, rockfish and naked mole rats (Finch, 2009), is intrinsically linked to a defiance of the Gompertz-Makeham law of mortality (Gompertz, 1825; Makeham, 1860), which states that death risk increases exponentially as an organism ages. Indeed, a recent study involving three salamander species (*Lyciasalamandra fazilae, Salamandra salamandra*, and *Salamandra perspicillata*), indicates that their mortality rate is stable and weakly affected by age, in keeping with them exhibiting negligible senescence (Cayuela et al., 2019). This observation raises several important yet outstanding questions, including whether salamanders manifest cellular hallmarks of ageing as defined in mammalian contexts (Lopez-Otin et al., 2013), whether they age at the molecular level, what principles govern ageing—or lack of*—*in these organisms and, in particular, what is the role played by their extreme regenerative abilities in this process.

As a salamander grows older, changes in its tissues do occur. A number of these have been reported for the axolotl, including increase in size, progressive replacement of skeletal cartilage by bone, reduced locomotion and thickening of the dermal layer (Vieira et al., 2020). However, these changes are likely associated with the species’ traits—as in the case of size-, and organism’s maturation—in the case of the skeleton, dermis and locomotion*—*rather than ageing. In addition, a reduction in the rate of limb regeneration through time has been observed for the axolotl (Vieira et al., 2020). Yet, this can be interpreted as a consequence of the continuous growth that characterises this species, as the regeneration rate is proportional to the size of the structure being regenerated. Furthermore, time-related declines in regeneration rate are not observed in salamander species with limited adult growth (e.g., *Notophthalmus viridescens*). An additional change that may occur in axolotls as they age is a decline in fertility. This notion, based on anecdotal reports of mating success, including in our colony, does not extend to other salamander species, nor is conserved across the amphibian clade (Jones et al., 2014).

Long lifespans combined with a lack of ageing biomarkers have so far precluded the determination of biological age in urodeles. While this issue has seldom been studied, time-related expression changes have been reported for aged tail and iris cells from c. 30 year old newts (Sousounis et al., 2015). Some of these changes are consistent with features of molecular ageing as observed in mammalian contexts. Namely, aged tail and iris samples displayed a downregulation of electron transport chain genes when compared to their young counterparts, indicating that these tissues could undergo molecular ageing (Sousounis et al., 2015). Nevertheless, further research should determine to what extent salamander tissues age at the molecular level.

## Cancer Resistance

Molecular ageing aside, no clear manifestations of age-related physiological declines or pathologies have been reported in urodeles to date. On the contrary, they exhibit a very low incidence of cancer, one of the most prevalent age-related pathologies. Neoplastic growth is rarely observed among salamander species, as documented in newts and axolotls (Ingram, 1971; Tsonis, 1983). Further, treatment with carcinogens can result in neoplasm induction but only at higher concentrations and longer treatment periods than those required to elicit malignant transformations in mammalian settings (Tsonis, 1983). Evidence also indicates that regenerating tissues such as limbs are particularly resistant to tumourigenesis (Tsonis and Eguchi, 1981). Indeed, malignant outgrowths show regression, incorporation to regenerating tissues or induction of axis duplications and accessory limbs, yet they do not persist as tumours in the regenerated structure (Breedis, 1952; Ingram, 1971). This is surprising as, paradoxically, the process of regeneration shares many similarities with tumour development, including downregulation of tumour suppressors (e.g., p.53, Yun et al., 2013, 2014), upregulation of oncogenes (e.g., c-myc, Maki et al., 2009), and extensive cell proliferation (Subiran Adrados et al., 2020). In line with Waddington’s individuation field hypothesis (Waddington, 1935) it is possible that, in a regenerative context, active patterning and differentiation mechanisms influence cell behaviour away from neoplasia. However, this is a notion that merits further consideration.

## Salamanders and the Hallmarks of Ageing

When looking for factors that may account for the absence of age-related declines in urodeles, it is worth considering whether and how hallmarks of ageing are manifested in these organisms. One such hallmark is cellular senescence, which in the recent years has emerged as a driver of several age-related disorders. Senescent cells are induced by various forms of cellular stress such as DNA damage, telomere shortening, oxidative challenges and oncogene activation (Gorgoulis et al., 2019). In response to these stimuli, these cells undergo a permanent cell cycle arrest and acquire a characteristic phenotype which includes the ability to secrete a repertoire of growth factors, matrix remodelling proteins and modulators of inflammation and immunity (Walters and Yun, 2020). Senescent cells play physiological roles in a number of contexts, including development (Munoz-Espin et al., 2013; Storer et al., 2013; Davaapil et al., 2017), wound healing (Jun and Lau, 2010; Demaria et al., 2014; Ritschka et al., 2017) and tissue repair processes (Yun et al., 2015; Sarig et al., 2019; Da Silva-Alvarez et al., 2020), in both mammals and salamanders. However, in mice and humans, they accumulate in various tissues as the organism ages, resulting in an imbalance in the inflammatory response and the promotion of age-related disorders such as sarcopenia, atherosclerosis, subcutaneal fat loss, osteoarthritis and neurodegeneration (van Deursen, 2014; Gorgoulis et al., 2019). Importantly, this role is causal, as the elimination of senescent cells attenuates age-related decay and leads to significant lifespan extension in mice (Baker et al., 2011, 2016). Moreover, it has been recently suggested that an age-related slow-down in senescent cell turnover could be a major contributing factor to the Gompertz law of mortality (Karin et al., 2019), which several salamander species defy. Relevant to this suggestion, we have observed that axolotls and newts (up to 10 years old) do not accumulate senescent cells in their tissues—e.g., liver, spleen, heart, limbs*—*as they age (Yun et al., 2015). Further, we have now extended these observations for axolotls up to 20 years old, and have not observed senescent cell accumulation. While the mechanistic reasons for this phenomenon remain elusive, it is notable that salamanders have a very rapid and efficient immune-dependent mechanism for senescent cell clearance, which may account for the lack of senescent cell accumulation (Yun et al., 2015; Walters and Yun, 2020). It is also possible that avoidance of replicative senescence, a form of senescent cell arrest triggered by telomere shortening, also plays a role in this context. While this process has not been studied in urodeles, observations suggest that salamander cells do not exhibit replicative senescence in culture (Ferretti and Brockes, 1988). Given the strong correlation between telomere shortening rate and the lifespan of a species (Whittemore et al., 2019), this is a topic worthy of further research efforts. In addition, another factor that could contribute to a lack of senescent cell accumulation in salamanders is the existence of well-geared mechanisms of genome maintenance. Although still a poorly developed area, recent studies suggest that axolotls employ robust DNA damage response mechanisms to promote proper cell cycle progression upon injury (Sousounis et al., 2020), which may restrict excessive generation of senescent cells in regenerative contexts. This is an interesting notion in light of evidence suggesting that other species of negligible senescence, such as the naked mole rat, exhibit efficient DNA repair mechanisms (Tian et al., 2017). In addition, it is possible that their large genomes (Elewa et al., 2017; Nowoshilow et al., 2018; Smith et al., 2019) provide an additional level of protection against mutagenic challenges, as the presence of extensive non-coding, non-regulatory areas would help ease the mutagenic burden (Poetsch et al., 2018). Nevertheless, it is yet unclear if the mechanisms of genome maintenance in salamander cells are more efficient than those found in their mammalian counterparts both in regenerative and homeostatic contexts, whether salamander cells are more resistant to certain types of genome challenges, and how well does this explain their limited senescent cell accumulation and resistance to age-related decay.

Another important hallmark with a well-documented impact on ageing and longevity is metabolic dysregulation. In particular, deregulated nutrient sensing is one of the most common age-related traits, with the insulin–insulin growth factor 1 (IGF1) signalling pathway constituting the most conserved age-controlling mechanism in evolution (Lopez-Otin et al., 2013; Fontana and Partridge, 2015; Partridge et al., 2018). This pathway mediates, partially through its control of the age-implicated mTOR complexes, the beneficial effects of dietary restriction on longevity from mice through worms to flies. Genetic mutations leading to a reduction in the functions of insulin receptor, IGF1 or mTOR result in lifespan extension (Fontana and Partridge, 2015). Further, mTOR inhibition through rapamycin leads to significant longevity increases, one of the most robust pharmacological interventions to promote lifespan extension in mammalian contexts (Fontana and Partridge, 2015). While little is known of how salamanders regulate nutrient sensing and its connection to their longevity, it is noteworthy that salamanders, as ectotherms, have inherently high levels of metabolic plasticity including thermal acclimation and hibernation/aestivation cycles, which may facilitate achieving metabolic states—at least temporarily*—*similar to those conducing to lifespan extension in mammals. Nevertheless, it is also worth noting that the aforementioned molecular regulators of anti-ageing are also involved in regenerative processes (Lund-Ricard et al., 2020). Particularly, IGF1 and mTOR inhibition suppress blastema formation during zebrafish fin regeneration (Hirose et al., 2014). In axolotls, mTORC1, a key complex whose downregulation promotes mammalian longevity, is implicated in mediating a systemic response to injury (Johnson et al., 2018). It would thus be of interest to understand the activity balance of this molecular axis in connection to both ageing and regeneration.

## On the Link Between Regeneration and Ageing

Together, the aforementioned observations raise a critical question, namely what is the link between longevity, lack of age-related decay and extreme regenerative abilities such as those found in salamanders? Could regeneration, in particular the limited reprogramming used by these organisms, elicit a process akin to tissue rejuvenation? Again, almost no studies to date have tackled this question. Anecdotal observations indicate that in axolotls the regenerated skin is structurally distinct from that of the original tissue, exhibiting greater thickness and dermal connective tissue (McCusker and Gardiner, 2011), suggesting a phenomenon akin to rejuvenation. Further, it has been proposed that the activation of developmental pathways in the regenerative context would lead to the generation of new tissues of equivalent age to those that arise immediately upon development (McCusker and Gardiner, 2011). While this idea has not been formally addressed, the study by Sousounis et al. has offered initial insights into this problem. By comparing gene expression signatures of lenses that had undergone repeated regeneration cycles to those of the original lenses, the authors observed that the regenerated lens transcriptome resembled the original one and thus appeared not to have aged. In contrast, old structures that never regenerated, such as the tail or the iris—the source of progenitors for the lens*—*exhibited more noticeable time-related changes (Sousounis et al., 2015). Unfortunately, this study did not include a comparison with old lenses that had never undergone regeneration, therefore its conclusions are based on the effect of time on other tissue populations. Consequently, the similarities in gene-expression between original and regenerated lenses could be explained by a rejuvenation effect, but also by an inherent resistance of the lens itself to the passing of time that is not found in tail or iris tissues. Whilst this remains an open question, this study constitutes a first attempt to address the link between the limited reprogramming associated to lens regeneration and rejuvenation. This is particularly interesting in light of recent findings suggesting that reprogramming may revert aging through epigenomic mechanisms (Lu et al., 2020). Challenging the link between regeneration and rejuvenation further will require establishing reliable aging biomarkers in salamanders, capable of accurate determinations of tissue age, and leveraging systems approaches to determine the molecular changes that occur with time from individual cells to entire structures and how these are affected by regenerative processes. Lastly, this would also benefit from comparative genomic approaches, taking advantage of the available repertoire of models of regeneration and ageing (Valenzano et al., 2017).

## Concluding Remarks

Salamanders offer a wealth of interesting biology, from their seemingly endless regenerative abilities to their extreme lifespans and resistance to cancer and age-related decay. Thanks to recent technological advances in transgenesis, gene editing tools and fully assembled genomes, models such as the axolotl and the Iberian ribbed newt provide an opportunity to unravel the cellular and molecular basis of these remarkable traits. Together, the resulting insights will help us further understand the nature of regeneration, ageing and their interconnection, central to the development of rejuvenation strategies of clinical relevance.

## Data Availability Statement

The original contributions presented in the study are included in the article/supplementary material, further inquiries can be directed to the corresponding author/s.

## Author Contributions

MHY conceived and wrote this manuscript.

## Conflict of Interest

The author declares that the research was conducted in the absence of any commercial or financial relationships that could be construed as a potential conflict of interest.
